# Association Between Symptoms of Common Mental Disorders and Adolescent Smoking: A Cross‐Sectional Study From Indonesia's Basic Health Research Survey

**DOI:** 10.1002/hsr2.73421

**Published:** 2026-10-26

**Authors:** Bagas Abdillah, Mintu Nath

**Affiliations:** ^1^ Institute of Applied Health Sciences University of Aberdeen Aberdeen UK; ^2^ Faculty of Health, Medicine and Life Sciences Universitas Airlangga Banyuwangi Indonesia

**Keywords:** adolescent smoking, common mental disorders, prevalence, survey data

## Abstract

**Background and Aims:**

Evidence on the association between common mental disorder symptoms and adolescent smoking in a large nationally representative sample remains limited. We investigated this relationship among Indonesian adolescents using national survey data.

**Methods:**

We analysed cross‐sectional data from the 2018 Indonesian Basic Health Research survey, a two‐stage stratified sample of 176,555 adolescents aged 10–18 years. Symptoms of common mental disorder were assessed in 70,969 adolescents aged 15–18 years using the Mini International Neuropsychiatric Interview (MINI) questionnaire and the Self‐Reporting Questionnaire‐20. We estimated the prevalence of adolescent smoking. We developed a directed acyclic graph (DAG) to identify the potential confounders and applied multivariable logistic regression models with survey weights to evaluate the association between common mental disorder symptoms and adolescent current smoking, controlling for measured confounders.

**Results:**

The prevalence of adolescent current smoking aged 10–18 years in Indonesia was 9.10% (95% CI: 9.03, 9.16). The prevalence of adolescent current smoking among those completing common mental disorder symptom assessment was 18.14% (95% CI: 18.00, 18.18). Both cohorts showed higher prevalence in male, older, and rural groups. Adolescents with symptoms of common mental disorder had increased odds of current smoking: estimated odds ratio of 1.59 (95% CI: 1.40, 1.81). Important measured confounders of the estimation obtained from DAG analysis and adjusted for in the final model were age, sex, current education, place of residence, working status, and health behaviours (alcohol use, physical activity, and dietary patterns).

**Conclusions:**

The prevalence of adolescent smokers in Indonesia is higher than reported previously. This study established the association between common mental disorder symptoms and adolescent current smoking. Incorporating mental disorder screening and support into prevention strategies could strengthen interventions and inform future policy to reduce the increased prevalence of youth smoking.

## 1 Introduction

Tobacco consumption among adolescents is a critical public health concern, as most adult smokers begin smoking during adolescence, leading to lifelong nicotine dependence and addiction [1]. Early initiation is particularly harmful: starting at a younger age increases the likelihood of premature death from smoking‐related adverse health conditions such as cardiovascular diseases, chronic obstructive pulmonary disease (COPD), and various cancers, alongside short‐term impacts like impaired lung development and reduced physical fitness [2].

Globally, an estimated 11.3% adolescents are current smokers [3]. While many countries reported a decline in prevalence over the years, adolescent smoking prevalence in Indonesia has remained persistently high, with national survey data showing an increase among youth aged 10–18 years from 7.2% in 2013 [4] to 9.1% in 2018 [5], while recent Global Youth Tobacco Survey (GYTS) data indicates that tobacco use reached 19.2% among school‐going adolescents [6]. This has prompted the Indonesian government to prioritise reducing smoking prevalence among adolescents aged 10–18 years, given its potential long‐term burden on adult health and the associated risk to the nation's demographic dividend projected between 2030 and 2045 [7].

In Indonesia, 28.9% of individuals aged ≥ 10 years are active smokers [5]. Most initiated smoking at a very young age, 23.1% started smoking at 10%–14% and 52.1% at 15–19 [5]. To control different factors, the government has implemented programmes targeting adolescents, including smoke‐free areas, excise taxes and cigarette price increases (sin tax), and bans on advertising, promotion, and sponsorship [7]. These interventions primarily target environmental factors. However, individual‐level determinants, particularly mental disorder, have received little attention, despite adolescence being a period of major neurological and psychological changes that may influence risk behaviours [8].

In psychiatric epidemiology, symptoms of mental disorders in non‐clinical populations are conceptualised as common mental disorders, representing a continuum of psychological distress, such as depression, anxiety, and somatic symptoms that impairs functioning without requiring a formal clinical psychiatric diagnosis [9, 10, 11]. There are several compelling evidence that mental health problems like depression, stress, and anxiety are positively associated with enhanced smoking among adolescents, including earlier initiation and increased consumption [12, 13, 14, 15, 16]. Such adolescents are also less likely to quit smoking and more prone to relapse in adulthood [17]. Evidence on nicotine's interaction with neurotransmitter pathways supports the hypothesis that mental disorders may increase susceptibility to smoking by reinforcing a cycle of use and dependence [18, 19]. This supports the hypothesis that the presence of symptoms of common mental disorders is associated with increased likelihood of current smoking among Indonesian adolescents. However, many prior studies have relied on small cohorts [13, 15, 16]. Relatively few studies have examined the association between symptoms of common mental disorder and current smoking using large‐scale population‐based datasets [12, 14], particularly considering the complexity of the relationship in the presence of multifaceted risk factors. In addition, most studies have focused on adult populations, limiting their generalisability to adolescents.

To address the research gap, the present study investigated the association between symptoms of common mental disorder and current smoking among adolescents. Using nationally representative data from the 2018 Indonesian Basic Health Research Survey, we estimated the prevalence of adolescent current smoking in the 10–18‐year age group. We then constructed a directed acyclic graph (DAG) to identify potential confounders and incorporated these into multivariable models to estimate the association of symptoms of common mental disorder with adolescent current smoking.

## 2 Methods

### 2.1 Data Source

This cross‐sectional study used secondary data from the 2018 Indonesian Basic Health Research (IBHR) survey, a nationally representative survey of the Indonesian population. The IBHR survey collected information on a broad range of health domains, including health services, communicable and non‐communicable diseases, nutrition, reproductive and maternal health, dental and oral health, disabilities and injuries, mental health, and behavioural risk factors. Data were collected through interviews, measurements, and examinations. The survey was conducted in April and May 2018 based on sampling in March 2018 [5].

### 2.2 Sampling Technique

The IBHR survey employed a two‐stage stratified sampling design [5]. In the first stage, from 720,000 census blocks across Indonesia, 180,000 (25%) were selected as the sampling frame using implicit stratification based on welfare strata. A census block is a small administrative unit representing a small part of a village, usually containing around 80–120 households. From this sampling frame, 30,000 census blocks were systematically selected using a probability proportional to size within each urban/rural stratum across districts and municipalities. In the second stage, ten households were selected from each census block using systematic sampling, stratified by the head of the household's education level. This yielded 300,000 households with all household members invited to participate in the survey.

In the smoking survey, the questionnaire was asked to respondents aged ≥ 10 years, and 818,507 individuals completed the survey. To estimate the prevalence of adolescent smoking, the present study considered 176,555 adolescent respondents aged 10–18 years [5]. Per the official 2018 IBHR survey protocol, common mental disorder symptom questionnaires (SRQ–20 and MINI) were administered exclusively to respondents aged 15 years and above, reflecting the validated age boundaries of these instruments in Indonesia. A total of 70,969 individuals aged 15–18 years completed the survey. This subset data represented the primary analytic cohort for evaluating the association between the presence of common mental disorder symptoms and current smoking. To evaluate potential sub‐sample selection bias, sociodemographic distributions of this analytic sub‐sample (15–18 years) were evaluated against the full adolescent sample (10–18 years).

The 2018 Indonesian Basic Health Research (IBHR) survey protocol was reviewed and ethically approved by the Health Research Ethics Committee of the Health Research and Development Agency (NIHRD/*Litbangkes*), Ministry of Health of the Republic of Indonesia (Ethical Approval Number: LB.02.01/2/KE.267/2017). Written informed consent was obtained from all adult participants and from parents/legal guardians for minor participants prior to data collection, along with informed assent obtained from the minor participants. This present study is a secondary analysis of the fully anonymized and de‐identified IBHR dataset. Official permission to access and use the dataset was granted by the Health Policy Agency (BKPK), Ministry of Health of the Republic of Indonesia (Data Permission Letter Number: IR.03.01/H.I/3663/2023). Because this study utilized non‐identifiable secondary data, additional institutional review board (IRB) approval was exempt.

### 2.3 Study Variables

The outcome, current smoking, was defined as having smoked any tobacco or nicotine products, including manufactured filter cigarettes, clove cigarettes (*kretek*), hand‐rolled cigarettes (*linting*), electronic cigarettes, and shisha/hookah within the 30 days prior to the survey, in accordance with World Health Organization (WHO) Global Youth Tobacco Survey (GYTS) measurement standards [6]. Respondents who had not smoked within the past 30 days were classified as non‐smokers (1 = current smoker, 0 = non‐smoker).

The exposure was the presence of common mental disorder symptoms (binary), measured as a composite variable based on the presence of depression symptoms (assessed via the Mini International Neuropsychiatric Interview [MINI]) or emotional mental disorder symptoms (assessed via the Self Reporting Questionnaire‐20 [SRQ‐20] with a national validated cutoff ≥6) [5, 9, 20, 21]. These measures reflected the respondent's mental state in the 2 weeks prior to the survey, capturing population‐level psychological distress rather than a formal clinical psychiatric diagnosis [10, 11]. Detailed operational definitions, coding procedures, and categorizations for all study variables are summarized in Supplementary Table 2.

Potential confounders were identified based on previous literature and mapped within the DAG conceptual framework. Demographic factors included sex (male or female), age (15, 16, 17, and 18 years), and place of residence (urban or rural), accounting for age‐graded smoking exposure, strongly gendered cultural norms in Indonesia, and geographic differences in tobacco accessibility [22, 23, 24]. Variables representing socioeconomic status were current education (five levels: not currently studying, elementary, junior high, senior high, and university) and working status (three levels: working, studying, not working or studying), capturing differential purchasing power and peer group exposures in academic versus workplace environments [25]. Behavioural and lifestyle covariates included physical activity (less, moderate, vigorous) based on the Global Physical Activity Questionnaire (GPAQ) calculation [26], dietary behaviour representing the amount and frequency of taking unhealthy foods and beverages (five levels: less or never, several times a month, several times a week, daily, and several times a day) according to the previous study categorisation [27], fruit and vegetable consumption (below or meets recommendation) based on WHO guidelines [28], and alcohol consumption (yes, no) based on the consumption of alcoholic drinks in the last 30 days. These lifestyle factors were included to account for broader health‐related behaviours and lifestyle patterns (lifestyle clustering) where health‐compromising habits co‐occur with psychological distress and substance use initiation [29, 30].

### 2.4 Data Analyses

We summarised all categorical variables as frequencies and percentages. The prevalence of adolescent current smoking and the corresponding 95% confidence intervals (CI) were estimated for the full dataset (10–18 years) and the subset (15–18 years) for the overall population, and stratified by sex, age group, and place of residence. Prevalence estimates were obtained using a logistic regression model accounting for the survey weights.

To identify and visualize potential confounding structures, we developed a qualitative, domain‐knowledge‐driven Directed Acyclic Graph (DAG) using the open‐source software *DAGitty* (http://dagitty.net/) [31]. DAG was employed strictly as a conceptual structural framework to guide variable selection based on directional separation (d‐separation) rules, rather than a quantitative model‐fitting procedure; thus, goodness‐of‐fit metrics were not computed. Applying d‐separation criteria identified the minimal sufficient adjustment set required to block open backdoor paths between common mental disorder symptoms and current smoking. This adjustment set comprised sociodemographic variables (sex, age, residence, education, working status) and lifestyle behaviours (alcohol consumption, physical activity, fruit/vegetable intake, and unhealthy food and beverage consumption). Unobserved theoretical factors, specifically genetic predisposition, were explicitly included as latent (unobserved) nodes in the DAG (represented by hatched circles and dashed biasing paths in Figure 1) to transparently acknowledge residual confounding inherent in secondary survey data.

**Figure 1:**
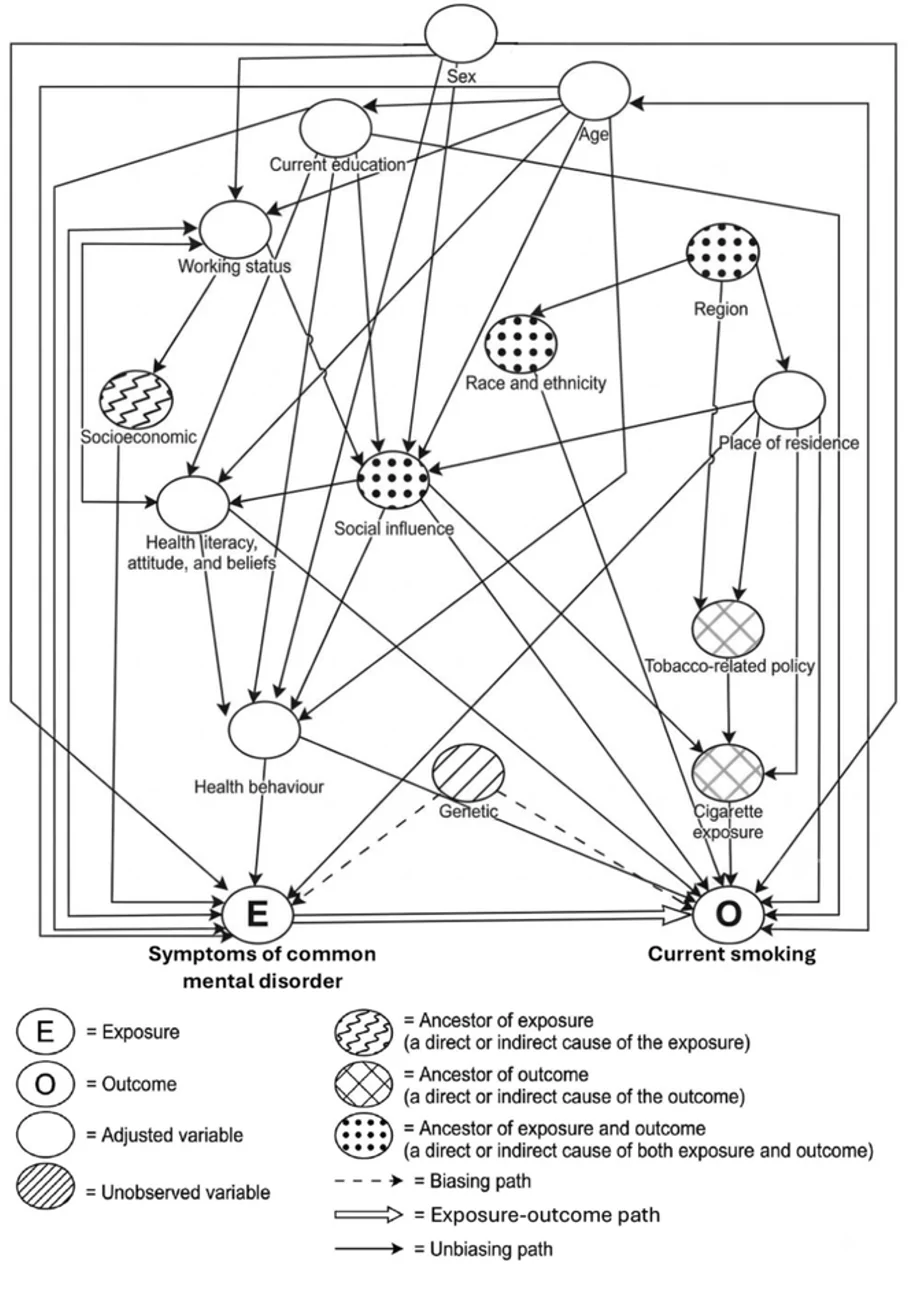
Directed Acyclic Graph (DAG) illustrating the assumed structural pathway linking common mental disorder symptoms (exposure) and adolescent current smoking (outcome). (Legend: Circles with patterns indicate ancestors of exposure/outcome; open circles represent adjusted variables; the hatched circle indicates an unobserved latent variable; dashed arrows represent biasing paths; solid arrows represent unbiasing paths). Confounding variables were identified as common causes of both exposure and outcome, including demographic, socioeconomic, and behavioural factors. *DAGitty* software identified two minimal sufficient adjustment sets, from which the more parsimonious set was selected to reduce reliance on unobserved variables. In the final model, observed confounders (age, sex, education, place of residence, working status, and health behaviours) were included in the analysis, while genetics remained as a latent or unobserved confounder.

We fitted survey‐weighted multivariable logistic regression models of symptoms of common mental disorder on current smoking, adjusting for the identified confounders. Analyses were restricted to the subset of respondents aged 15–18 years who completed the common mental disorder questionnaire (*n* = 70,969). Missing data across the exposure, outcome, and covariates was negligible (< 0.003%, n < 20); thus, a complete case analysis approach was applied, excluding observations with missing values. Odds ratios (ORs) and 95% CIs were reported. All statistical tests were two‐sided with a type I error rate of 5%.

All data management and statistical analysis were conducted in the R Statistical Software environment (version 4.4.3). The *survey* package was used for fitting weighted logistic regression models, and the *emmeans* package was used to estimate the marginal means from the fitted model. The present study is reported based on the guidelines provided by the Consensus‐based Checklist for Reporting of Survey Studies (CROSS) [32], and the checklist is summarised in Supplementary Table 1.

## 3 Results

### 3.1 Summary Statistics

A total of 176,555 adolescents aged 10–18 years were included to estimate the overall national prevalence of current smoking. Supplementary Table 3 summarises the sociodemographic characteristics of this full adolescent sample. From the full dataset, an analytic sub‐sample of 70,969 respondents aged 15–18 years who completed the common mental disorder questionnaires was selected as the analytic sample for further modelling; their baseline characteristics are detailed in Table 1. Within this subset, a proportion of adolescents reported symptoms indicative of common mental disorders.

**Table 1:** Summary statistics of subset dataset representing the responders completing common mental disorder questionnaire in the 2018 Indonesian Basic Health Research (IBHR) survey.

Characteristic	Current smoker (*N* = 11,820)		Non‐smoker (*N* = 59,149)		Total (*N* = 70,969)		
	*n*	%	*n*	%	*n*	%	
The presence of mental disorder symptoms						
Yes	1,405	11.9	6,519	11.0	7,924	11.2	
No	10,415	88.1	52,630	89.0	63,045	88.8	
Sex							
Male	11,664	98.7	24,814	42.0	36,478	51.6	
Female	156	1.3	34,335	58.0	34,491	48.6	
Age							
15–16 years	4,421	37.4	32,377	54.7	36,798	51.9	
17–18 years	7,399	62.6	26,772	45.3	34,171	48.1	
Place of residence							
Rural	7,017	59.4	32,911	55.6	39,928	56.3	
Urban	4,803	40.6	26,238	44.4	31,041	43.7	
Working status							
Working	2,712	22.9	4,130	7.0	6.842	9.6	
Studying	5,804	49.1	39,957	67.6	45,761	64.5	
Not working or studying	3,304	28.0	15,062	25.5	18,366	25.9	
Current education							
Not currently studying	6,019	50.9	19,216	32.5	25,235	35.6	
University	499	4.2	3,408	5.8	3,907	5.5	
Senior high school	4,026	34.1	25,988	43.9	30,014	42.3	
Junior high school	1,147	9.7	9,363	15.8	10,510	14.8	
Elementary school	129	1.1	1,174	2.0	1,303	1.8	
Fruit and vegetable consumption							
Below recommendation	11,433	96.7	57,101	96.5	68,534	96.6	
Meets recommendation	387	3.3	2,048	3.5	2,435	3.4	
Unhealthy food consumption							
Several times a day	9,932	84.0	48,318	81.7	58,250	82.1	
Daily	1,516	12.8	8,565	14.5	10,081	14.2	
Several times a week	257	2.2	1,537	2.6	1,794	2.5	
Several times a month	109	0.9	627	1.1	736	1.0	
Less or never	6	0.1	102	0.2	108	0.2	
Unhealthy beverage consumption							
Several times a day	1,472	12.5	3,023	5.1	4,495	6.3	
Daily	5,609	47.5	21,004	35.5	26,613	37.5	
Several times a week	3,001	25.4	20,588	34.8	23,589	33.2	
Several times a month	1,503	12.7	11,435	19.3	12,938	18.2	
Less or never	235	2.0	3,099	5.2	3,334	4.7	
Alcohol consumption							
Yes	2,112	17.9	582	1.0	2,694	3.8	
No	9,708	82.1	58,567	99.0	68,275	96.2	
Physical activity							
Less	4,997	42.3	27,739	46.9	32,736	46.1	
Moderate	471	4.0	5,031	8.5	5,502	7.8	
Vigorous	6,352	53.7	26,379	44.6	32,731	46.1	

### 3.2 Prevalence of Adolescent Smoking

Table 2 summarises the prevalence of adolescent smokers in Indonesia. The estimated prevalence of current smoking among Indonesian adolescents aged 10–18 years was 9.10% (95% CI: 9.03–9.16). Smoking prevalence was markedly higher among males (17.48%, 95% CI: 17.09–17.87) than females (0.25%, 95% CI: 0.20–0.31). Prevalence increased with age, from 0.58% (95% CI: 0.51–0.68) at 10–12 years to 22.83% (95% CI: 22.16–23.52) at 17–18 years. Adolescents in rural areas reported a slightly higher prevalence (9.48%, 95% CI: 9.21–9.75) than those in urban areas (8.76%, 95% CI: 8.44–9.09). In the subset dataset (15–18 years), smoking prevalence was higher at 18.14% (95% CI: 18.00–18.28), consistent with age‐related increases observed in the full dataset.

**Table 2:** Prevalence of current smoking among indonesian adolescents.

Characteristics	Full data (*n* = 176,555)			Subset data (*n* = 70,969)		
	Prevalence	95% CI		Prevalence	95% CI	
		LCI	UCI		LCI	UCI
Overall	9.10	9.03	9.16	18.14	18.00	18.28
Sex						
Male	0.25	0.20	0.31	0.50	0.40	0.63
Female	17.48	17.09	17.87	34.86	34.12	35.61
Age						
10–12 years	0.58	0.51	0.68	—	—	—
13–14 years	4.45	4.16	4.76	—	—	—
15–16 years	13.39	12.85	13.95	13.48	12.93	14.04
17–18 years	22.83	22.16	23.52	22.91	22.23	23.59
Place of residence						
Rural	9.48	9.21	9.75	19.14	18.59	19.70
Urban	8.76	8.44	9.09	17.31	16.66	17.98

*Note:* The full data represents participants aged 10 years and above who completed the smoking survey questionnaire. The subset data represents participants aged 15 years and above who responded to the common mental disorder questionnaires.

### 3.3 Confounder Identification

To identify potential confounders in the pathway between symptoms of common mental disorder and current smoking, we employed a directed acyclic graph (DAG). In DAG terminology, a confounder is defined as a common cause of both the exposure and the outcome [31]. Using *DAGitty* software two alternative minimal adjustment sets were identified to estimate the total effect of common mental disorder symptoms on current smoking. The first set included age, sex, current education, place of residence, working status, race/ethnicity, social influence, tobacco‐related policy, genetics, health behaviours, and attitudes/beliefs. The second, more parsimonious set included age, sex, current education, place of residence, working status, genetics, and health behaviours (included as an observed representation of health literacy, attitudes and beliefs) (Figure 1).

We selected the second parsimonious adjustment set because it involved fewer unobserved (latent) variables. To address the potential confounding effect of latent constructs such as health literacy and attitudes/beliefs, we used proxy variables (e.g., health behaviours) that are strongly correlated with these constructs. It is expected that a surrogate variable strongly associated with an unobserved confounding variable would remove a large part of the unobserved confounding [33]. These adjustment sets effectively blocks all identified backdoor biasing paths while preserving exposure‐outcome path [31]. Consequently, genetics remained the sole unmeasured (latent) structural confounder in the final theoretical model.

### 3.4 Association Between Symptoms of Common Mental Disorder and Adolescent Current Smoking

Figure 2 and Supplementary Table 4 show the association between symptoms of common mental disorder and current smoking in the subset of adolescents aged 15–18 years. Adolescents with symptoms of common mental disorder had significantly higher odds of current smoking compared with those without symptoms (aOR: 1.59, 95% CI: 1.40–1.81). The model adjusted for several confounding variables like sex, age, place of residence, current education, working status, and health behaviours (alcohol and food consumption, fruit and vegetable intake, and physical activity). Some confounders also showed strong associations with current smoking. Male adolescents were far more likely to smoke than females (aOR: 108.16, 95% CI: 86.04–135.96), reflecting the very low prevalence among females. Current smoking likelihood increased with age and was higher in rural than in urban areas. Adolescents who were working were more likely to smoke, while those still studying were less likely to smoke compared with those not working or studying. Unhealthy behaviours, including frequent consumption of unhealthy foods and beverages, low fruit and vegetable intake, and alcohol use, were associated with greater odds of current smoking.

**Figure 2:**
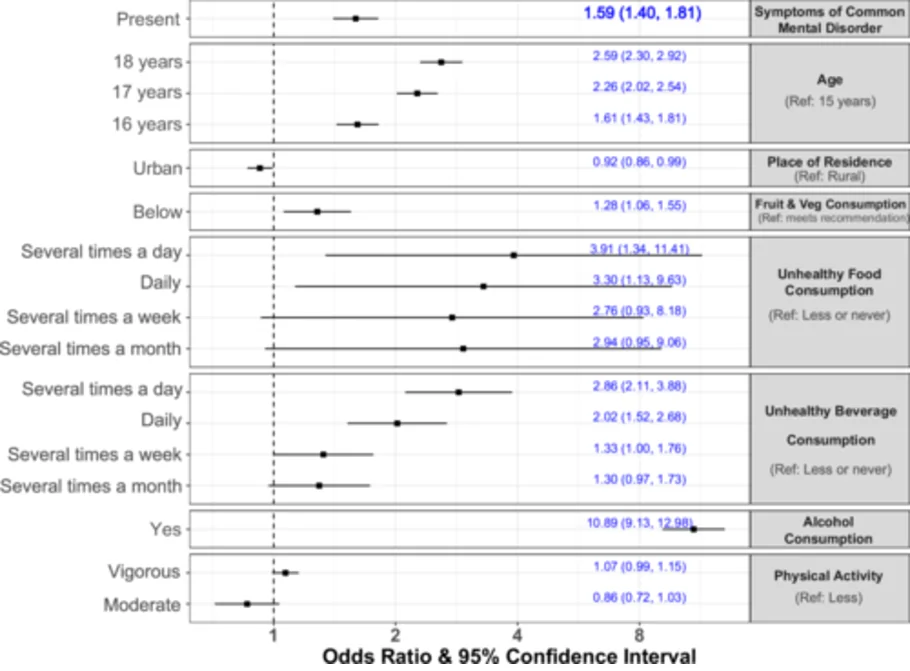
Estimates of odds ratio (points) and corresponding 95% confidence interval (error bars) of categories of predictors (compared to the reference category) from the fitted multivariable logistic regression model of current smoking status in adolescents aged 15–18 in a subset dataset aged 15–18 who had completed the common mental disorder symptom questionnaire (*n* = 70,969). The x‐axis is on the log_2_ scale. The confidence intervals that do not include the dotted vertical line are statistically significant (*p* < 0.05).

## 4 Discussion

Using the largest nationally representative survey dataset on the adolescent population in Indonesia, we estimated the prevalence of current smoking among those aged 10–18 at 9.10%. Male, older age, and rural residence were associated with higher current smoking prevalence. Adolescents reporting symptoms of common mental disorder had significantly higher odds of current smoking (OR: 1.59; 95% CI: 1.40–1.81), even after adjusting for confounders identified through DAG analysis. Smokers were also more likely to engage in other unhealthy behaviours, including alcohol use, consumption of unhealthy foods and beverages, and low fruit and vegetable intake.

Our study demonstrated an increase in adolescent current smoking in Indonesia, from 7.2% in 2013 [4] to 9.1% in 2018 (this study). Consistent with prior reports, Indonesia ranks among the countries with the highest prevalence of adolescent smoking worldwide and is one of the few countries where prevalence has not declined since 1999, along with Botswana, Zimbabwe, Bosnia, and Herzegovina [3]. Within the Southeast Asia region, Indonesia has the second‐highest prevalence of adolescent smoking after Timor Leste [34].

Prior studies reported positive associations between mental health problems such as depression, stress, or anxiety and adolescent smoking, including earlier initiation and heavier consumption [16, 35, 36]. National surveys in other contexts have similarly shown that adolescents with symptoms of mental disorder are more likely to initiate smoking, progress to daily use, and smoke more heavily [15]. Mental distress in adolescence reduces the likelihood of quitting smoking and increases relapse risk in adulthood [17]. Adolescents with asthma and comorbid depression and anxiety, following adjustment of potential demographic and clinical covariates, were more likely to be smokers and had increased susceptibility to initiation of smoking [37]. A study of common mental disorder symptomatology before and during the COVID‐19 pandemic also supported the finding that after adjusting for sociodemographic factors, increased anxiety and depression symptoms increased the likelihood of cigarette and e‐cigarette consumption [38]. A longitudinal study using 16 years of nationally representative data applied robust econometric methods and concluded that improved mental health reduced the likelihood of smoking [39].

Our study confirms that common mental disorder symptoms are independently associated with adolescent current smoking. These findings strengthen the view that symptoms of mental disorder may contribute to smoking status, while acknowledging potential endogeneity from reverse causality, unmeasured confounders, and measurement error [39]. However, the possibility of a bidirectional relationship between smoking and mental health symptoms has been discussed in previous studies [40, 41]. Reviews suggesting bidirectionality were largely based on studies conducted in high‐income settings, predominantly the United States, with relatively small sample sizes and substantial heterogeneity in study design and outcome definitions. In addition, several studies did not adequately adjust for important confounders, such as alcohol consumption and other health‐related behaviours. Hence, evidence supporting a clear causal direction from smoking to common mental disorder remains limited.

Biological plausibility further supports this direction of association. Self‐medication theory proposes that individuals with psychological distress smoke to alleviate symptoms or to facilitate coping with their mental illness [42]. Nicotine stimulates nicotinic acetylcholine receptors, releasing dopamine and other neurotransmitters involved in reward and stress regulation [43, 44]. However, these effects contribute to short‐term coping behaviour, which may provide short‐term relief from stress and depression but reinforce dependence on smoking, tending to self‐medicate [45]. Tobacco comprises various compounds, notably nicotine, which could elicit the release of various substances such as norepinephrine, serotonin, dopamine, acetylcholine, γ‐aminobutyric acid, and glutamate in the brain that can relieve stress, anxiety, and depression, but prolonged use increases the risk of addiction and may worsen vulnerability to depression and anxiety [36].

Our study also confirmed sociodemographic patterns: current smoking was more common among males, older adolescents, and those in rural areas [22]. In Indonesia, smoking is strongly tied to cultural constructions of masculinity [23], while it is considered taboo or morally flawed in females [46]. Rural populations tend to have higher acceptance of smoking, which may explain urban–rural differences. Older adolescents may be more exposed to peers and social pressures, financially independent and have easier access to cigarettes [22, 24]. Employment also increased smoking risk, likely due to exposure to smoking peers and financial independence, while students were less likely to smoke due to restrictions in educational environments [25].

Our approach of a robust inferential framework, combined with a DAG to guide confounder selection, strengthens the validity of our findings. From a policy perspective, these results have practical relevance. Adolescent smoking is a complex, multifactorial behaviour that requires coordinated action at individual, family, school, and community levels. Governments could therefore develop comprehensive policies and programmes that enable parents, schools, and community services to work collaboratively, building integrated interventions that foster supportive environments and promote both mental health and healthy behaviours. Embedding such approaches within healthcare and allied settings may help prevent escalation of adverse smoking addiction and the exacerbation of health issues in adolescents. Such integrated models have been implemented in Australia and the United States and have been reported as successful [47, 48]. Collaboration and integration would help identify and resolve this issue at the earliest opportunity, reducing longer‐term impacts as adolescents transition into adulthood.

This study has established the association of common mental disorder symptoms and current smoking in Indonesian adolescents, harnessing nationally representative cross‐sectional data. Nevertheless, the study has some limitations. First, although the dataset is large and nationally representative, it is cross‐sectional; exposure and outcome were measured simultaneously, meaning temporal sequence, directionality, and potential reverse causality cannot be established. Second, current smoking status was self‐reported, raising the possibility of underreporting, particularly among female adolescents. Third, common mental disorder was assessed using screening instruments (MINI and SRQ‐20), which capture symptoms but do not establish clinical diagnoses. Fourth, some relevant confounders such as family history (e.g., parental smoking), genetic predisposition, social influence, individual temperament, and health literacy were unobserved in the dataset, while broader cultural nuances were only approximated using socioeconomic and demographic variables.

To summarise, the prevalence of adolescent current smoking in Indonesia is higher than that reported earlier. Common mental disorder symptoms were associated with a 59% increase in the odds of current smoking among adolescents (OR: 1.59, 95% CI: 1.40–1.81). By providing nationally representative empirical evidence from a high‐burden LMIC setting, this study extends existing literature beyond traditional environmental models. Future tobacco‐control policies should integrate mental disorder screening among the adolescent population and support into prevention strategies, alongside existing environmental measures, to more effectively reduce adolescent smoking.

## Author Contributions

**Bagas Abdillah:** conceptualization, methodology, software, data curation, investigation, validation, formal analysis, visualization, writing – original draft, writing – review and editing. **Mintu Nath:** conceptualization, methodology, software, investigation, validation, formal analysis, supervision, visualization, writing – review and editing.

## Ethics Statement

Informed consent was obtained during data collection, which considered aspects of the data collection procedure, the voluntary nature, and confidentiality. The anonymous data were shared with the authors by the Indonesian Government (permission number: IR.03.01/H.I/3663/3023).

## Conflicts of Interest

The authors declare no conflicts of interest.

## Transparency Statement

The lead authors Bagas Abdillah and Mintu Nath affirm that this manuscript is an honest, accurate, and transparent account of the study being reported; that no important aspects of the study have been omitted; and that any discrepancies from the study as planned (and, if relevant, registered) have been explained.

## Supporting information


Supporting File

## Data Availability

The data used in this study was granted by the Health Policy Agency of the Ministry of Health of the Republic of Indonesia, which can be accessed with specific requirements and procedures through www.badankebijakan.kemkes.go.id.
