# Supplementary material for: Association Between Symptoms of Common Mental Disorders and Adolescent Smoking: A Cross‐Sectional Study From Indonesia's Basic Health Research Survey
Source: Health Sci Rep. 2026 Oct 26;9(11):e73421. doi: 10.1002/hsr2.73421 (PMC13697518; doi:10.1002/hsr2.73421)
Supplement: Supplementary file 1 [file HSR2-9-e73421-s001.pdf]

Supplementary Material:

Association between symptoms of common mental disorders and adolescent smoking: A cross-sectional study from Indonesia's Basic Health Research Survey

Contents

Supplementary Table 1. Checklist for Reporting of Survey Studies (CROSS) 2

Supplementary Table 2. Detailed operational definitions, coding procedures, and categorizations for all study variables 6

Supplementary Table 3. Summary statistics of full dataset representing participants aged 10 years and above who completed the smoking behaviour survey questionnaire 9

Supplementary Table 4. Estimates of odds ratio (OR) and corresponding 95% confidence interval (95% lower, upper CI) from adjusted (multivariable) logistic regression model to evaluate the causal association between the presence of common mental disorder symptoms and risk of current smoking among adolescents in Indonesia 11

References 13

Supplementary Table 1. Checklist for Reporting of Survey Studies (CROSS)

Section/topic	Item	Item description	Reported on section (para)
**Title and abstract**			
Title and abstract	1a	State the word "survey" along with a commonly used term in title or abstract to introduce the study's design.	Yes
	1b	Provide an informative summary in the abstract, covering background, objectives, methods, findings/results, interpretation/discussion, and conclusions.	Yes
**Introduction**			
Background	2	Provide a background about the rationale of study, what has been previously done, and why this survey is needed.	1 (3,4,5)
Purpose/aim	3	Identify specific purposes, aims, goals, or objectives of the study.	1 (5)
**Methods**			
Study design	4	Specify the study design in the methods section with a commonly used term (e.g., cross-sectional or longitudinal).	2.1 (1)
Data collection methods	5a	Describe the questionnaire (e.g., number of sections, number of questions, number and names of instruments used).	2.3 (1,2,3)
	5b	Describe all questionnaire instruments that were used in the survey to measure particular concepts. Report target population, reported validity and reliability information, scoring/classification procedure, and reference links (if any).	2.3 (1,2,3)
	5c	Provide information on pretesting of the questionnaire, if performed (in the article or in an online supplement). Report the method of pretesting, number of times questionnaire was pre-tested, number and demographics of participants used for pretesting, and the level of similarity of demographics between pre-testing participants and sample population.	2.3 (3)
	5d	Questionnaire, if possible, should be fully provided (in the article, or as appendices or as an online supplement).	Data statement
Sample characteristics	6a	Describe the study population (i.e., background, locations, eligibility criteria for participant inclusion in survey, exclusion criteria).	2.2 (1,2)

Section/topic	Item	Item description	Reported on section (para)
	6b	Describe the sampling techniques used (e.g., single stage or multistage sampling, simple random sampling, stratified sampling, cluster sampling, convenience sampling). Specify the locations of sample participants whenever clustered sampling was applied.	2.2 (1)
	6c	Provide information on sample size, along with details of sample size calculation.	2.2 (1)
	6d	Describe how representative the sample is of the study population (or target population if possible), particularly for population-based surveys.	2.2 (1)
Survey administration	7a	Provide information on modes of questionnaire administration, including the type and number of contacts, the location where the survey was conducted (e.g., outpatient room or by use of online tools, such as SurveyMonkey).	2.2 (1)
	7b	Provide information of survey's time frame, such as periods of recruitment, exposure, and follow-up days.	2.2 (1)
	7c	Provide information on the entry process:<br>–>For non-web-based surveys, provide approaches to minimize human error in data entry.<br>–>For web-based surveys, provide approaches to prevent "multiple participation" of participants.	Not applicable
Study preparation	8	Describe any preparation process before conducting the survey (e.g., interviewers' training process, advertising the survey).	2.2 (1)
Ethical considerations	9a	Provide information on ethical approval for the survey if obtained, including informed consent, institutional review board [IRB] approval, Helsinki declaration, and good clinical practice [GCP] declaration (as appropriate).	2.2 (3)
	9b	Provide information about survey anonymity and confidentiality and describe what mechanisms were used to protect unauthorized access.	2.2 (3)
Statistical analysis	10a	Describe statistical methods and analytical approach. Report the statistical software that was used for data analysis.	2.4 (1,2,3,4)

Section/topic	Item	Item description	Reported on section (para)
	10b	Report any modification of variables used in the analysis, along with reference (if available).	2.3 (1,2,3)
	10c	Report details about how missing data was handled. Include rate of missing items, missing data mechanism (i.e., missing completely at random [MCAR], missing at random [MAR] or missing not at random [MNAR]) and methods used to deal with missing data (e.g., multiple imputation).	2.4 (3)
	10d	State how non-response error was addressed.	2.4 (3)
	10e	For longitudinal surveys, state how loss to follow-up was addressed.	NA
	10f	Indicate whether any methods such as weighting of items or propensity scores have been used to adjust for non-representativeness of the sample.	2.4 (3)
	10g	Describe any sensitivity analysis conducted.	2.4 (3)
**Results**			
Respondent characteristics	11a	Report numbers of individuals at each stage of the study. Consider using a flow diagram, if possible.	3.1 (1)
	11b	Provide reasons for non-participation at each stage, if possible.	3.1 (1)
	11c	Report response rate, present the definition of response rate or the formula used to calculate response rate.	3.1 (1)
	11d	Provide information to define how unique visitors are determined. Report number of unique visitors along with relevant proportions (e.g., view proportion, participation proportion, completion proportion).	NA
Descriptive results	12	Provide characteristics of study participants, as well as information on potential confounders and assessed outcomes.	3.2 (1)
Main findings	13a	Give unadjusted estimates and, if applicable, confounder-adjusted estimates along with 95% confidence intervals and p-values.	3.2 (1), 3.4 (1)
	13b	For multivariable analysis, provide information on the model building process, model fit statistics, and model assumptions	3.3 (1,2)

Section/topic	Item	Item description	Reported on section (para)
		(as appropriate).	
	13c	Provide details about any sensitivity analysis performed. If there are considerable amount of missing data, report sensitivity analyses comparing the results of complete cases with that of the imputed dataset (if possible).	NA
**Discussion**			
Limitations	14	Discuss the limitations of the study, considering sources of potential biases and imprecisions, such as non-representativeness of sample, study design, important uncontrolled confounders.	4 (9)
Interpretations	15	Give a cautious overall interpretation of results, based on potential biases and imprecisions and suggest areas for future research.	4 (9)
Generalizability	16	Discuss the external validity of the results.	4 (8)
**Other sections**			
Role of funding source	17	State whether any funding organization has had any roles in the survey's design, implementation, and analysis.	Yes
Conflict of interest	18	Declare any potential conflict of interest.	Yes
Acknowledgements	19	Provide names of organizations/persons that are acknowledged along with their contribution to the research.	Yes

Supplementary Table 2. Detailed operational definitions, coding procedures, and categorizations for all study variables

Variable	Type	Operational Definition & Coding Logic	Source
Current smoking (outcome)	Binary	0 = Non-smoker (did not smoke in the last 30 days)<br><br>1 = Current smoker (smoked tobacco daily or non-daily in the last 30 days)	[1]
Symptoms of Common Mental Disorder	Binary	A composite variable indicating the presence of common mental disorder symptoms, defined as having either depression symptoms or emotional mental disorder symptoms.<br><br>Composite assessment combining:<br><br>1. Mini International Neuropsychiatric Interview (MINI): Evaluated based on conditions experienced in the last 2 weeks. Categorized as **Present** if the respondent gives ≥ 2 "Yes" answers to core symptom questions (C01–C03) **AND** ≥ 2 "Yes" answers to additional symptom questions (C04–C010)<br><br>2. Self Reporting Questionnaire-20 (SRQ-20): Evaluated using the 20-item screening questionnaire. Categorized as Present if the total symptom score meets or exceeds the established nationally validated cut-off (≥ 6)<br><br>Present/Yes: Identified with depression symptoms (via MINI) OR emotional mental disorder symptoms (via SRQ-20)<br><br>Absent/No: Negative for both depression and emotional mental disorder symptoms	[2–5]
Sex	Binary	0 = Female<br><br>1 = Male	[4]
Age	Categorical	Single-year age of the respondent at the time of the survey:<br><br>15 = 15 years old<br><br>16 = 16 years old<br><br>17 = 17 years old	[4]

		18 = 18 years old	
Place of residence	Binary	0 = Rural<br><br>1 = Urban	[4]
Current education	Categorical	Education level currently enrolled:<br><br>1 = Not currently studying<br><br>2 = Elementary<br><br>3 = Junior high<br><br>4 = Senior high<br><br>5 = University	[4]
Working status	Categorical	Main activity in the past week:<br><br>1 = Not working or studying<br><br>2 = Studying (student)<br><br>3 = Working (employed / self-employed)	[4]
Fruit and vegetable consumption	Binary	Daily intake of combined fruits and vegetables evaluated against standard WHO dietary guidelines (≥ 400 grams or 5 standard portions/servings per day):<br><br>0 = Below recommendation / Inadequate (< 5 portions per day or < 400 g/day)<br><br>1 = Meets recommendation / Adequate (≥ 5 portions per day or ≥ 400 g/day)	[6]
Unhealthy food consumption	Categorical	Composite score derived from 5 food items: sweet food, salty food, fatty food, food with flavour enhancer, and instant food.<br><br>Original 6-point frequency categories were annualized: *Never* (0), *<3 times/month* (30), *1–2 times/week* (78), *3–6 times/week* (234), *1 time/day* (360), and >*1 time/day* (540 times/year), then divided by 52 to obtain average weekly frequencies. Weekly scores of the 5 items were summed and categorized into 5 levels:<br><br>1 = Less or never (< 0.75 times/week)<br><br>2 = Several times a month (0.75 to < 4 times/week)	[7]

		3 = Several times a week (4 to < 7 times/week)<br><br>4 = Daily (7 to < 14 times/week)<br><br>5 = Several times a day (≥ 14 times/week)	
Unhealthy beverage consumption	Categorical	Composite score derived from 3 beverage items: sweet beverages, soft drinks, and energy drinks.<br><br>Original 6-point frequency categories were annualized: *Never* (0), *<3 times/month* (30), *1–2 times/week* (78), *3–6 times/week* (234), *1 time/day* (360), and *>1 time/day* (540 times/year), then divided by 52 to obtain average weekly frequencies. Weekly scores of the 3 items were summed and categorized into 5 levels:<br><br>1 = Less or never (< 0.75 times/week)<br><br>2 = Several times a month (0.75 to < 4 times/week)<br><br>3 = Several times a week (4 to < 7 times/week)<br><br>4 = Daily (7 to < 14 times/week)<br><br>5 = Several times a day (≥ 14 times/week)	[7]
Alcohol consumption	Binary	0 = No (did not consume alcoholic beverages in the last 30 days)<br><br>1 = Yes (consumed alcoholic beverages in the last 30 days)	[4]
Physical activity	Categorical	Computed from 3 domains (work, transport, and recreation) using standard WHO MET assignments (Vigorous intensity = 8.0 METs; Moderate intensity & Transport = 4.0 METs).<br><br>Total physical activity was calculated as total MET-minutes/week across all domains and categorized into 3 levels based on WHO GPAQ criteria:<br><br>1 = Less / Low (< 600 MET-minutes/week)<br><br>2 = Moderate (600 to < 3,000 MET-minutes/week)<br><br>3 = Vigorous / High (≥ 3,000 MET-minutes/week)	[8]

Supplementary Table 3. Summary statistics of full dataset representing participants aged 10 years and above who completed the smoking behaviour survey questionnaire

Characteristic	Smoker (N = 13,900)		Non-smoker (N = 162,655)		Total (N = 176,555)	
	n	%	n	%	n	%
**Sex**						
Male	13,707	98.6	77,758	47.8	91,465	51.8
Female	193	1.4	84,897	52.2	85,090	48.2
**Age**						
10–12	358	2.6	63,457	39.0	63,815	36.1
13–14	1,668	12.0	39,241	24.1	40,909	23.2
15–16	4,459	32.1	32,998	20.3	37,457	21.2
17–18	7,415	53.3	26,959	16.6	34,374	19.5
**Place of residence**						
Rural	8,317	59.8	95,433	58.7	103,750	58.8
Urban	5,583	40.2	67,222	41.3	72,805	41.2
**Working status**						
Working	2,837	20.4	4,925	3.0	7,762	4.4
Studying	7,258	52.2	120,227	73.9	127,485	72.2
Not working or studying	3,805	27.4	37,503	23.1	41,308	23.4
**Current education**						
Not currently studying	6,645	47.8	42,452	26.1	49,097	27.8
University	499	3.6	3,423	2.1	3,922	2.2
Senior high school	4,153	29.9	29,145	17.9	33,298	18.9
Junior high school	2,149	15.5	43,351	26.7	45,500	25.8
Elementary school	454	3.3	44,284	27.2	44,738	25.3
**Fruit and vegetable consumption**						
Below recommendation	13,453	96.8	157,342	96.7	170,795	96.7
Meets recommendation	447	3.2	5,313	3.3	5,760	3.3
**Unhealthy food consumption**						
Several times a day	11,732	84.4	133,707	82.2	145,439	82.4
Daily	1,743	12.5	22,939	14.1	24,682	14.0
Several times a week	294	2.1	4,062	2.5	4,356	2.5

Characteristic	Smoker (N = 13,900)		Non-smoker (N = 162,655)		Total (N = 176,555)	
	n	%	n	%	n	%
Several times a month	122	0.9	1,674	1.0	1,796	1.0
Less or never	9	0.1	273	0.2	282	0.2
**Unhealthy beverage consumption**						
Several times a day	1,719	12.4	7,762	4.8	9,481	5.4
Daily	6,589	47.4	59,911	36.8	66,500	37.7
Several times a week	3,523	25.3	59,495	36.6	63,018	35.7
Several times a month	1,785	12.8	28,224	17.4	30,009	17.0
Less or never	284	2.0	7,263	4.5	7,547	4.3
**Alcohol consumption**						
Yes	2,303	16.6	830	0.5	3,133	1.8
No	11,597	83.4	161,825	99.5	173,422	98.2
**Physical activity**						
Less	6,068	43.7	91,562	56.3	97,630	55.3
Moderate	585	4.2	13,726	8.4	14,311	8.1
Vigorous	7,247	52.1	57,367	35.3	64,614	36.6

Supplementary Table 4. Estimates of odds ratio (OR) and corresponding 95% confidence interval (95% lower, upper CI) from adjusted (multivariable) logistic regression model to evaluate the causal association between the presence of common mental disorder symptoms and risk of current smoking among adolescents in Indonesia

Variables	OR	95% CI		P-value
		LCI	UCI	
**Common mental disorder symptoms**				
Yes	1.59	1.40	1.81	<0.001
**Sex**				
Male	108.16	86.04	135.96	<0.001
**Age (ref: 15 years)**				
16 years	1.61	1.43	1.81	<0.001
17 years	2.26	2.02	2.54	<0.001
18 years	2.59	2.30	2.92	<0.001
**Place of residence**				
Urban	0.93	0.86	0.99	0.03
**Working status (ref: Not working or studying)**				
Studying	0.13	0.03	0.66	0.01
Working	2.13	1.89	2.40	<0.001
**Current education (ref: Not currently studying)**				
Elementary school	4.21	0.83	21.35	0.08
Junior high school	4.69	0.94	23.32	0.06
Senior high school	4.92	0.99	24.37	0.05
University	4.71	0.95	23.50	0.06
**Fruit and vegetable consumption (ref: Meets recommendation)**				
Below	1.28	1.06	1.55	0.01
**Unhealthy food consumption (ref: Less or never)**				
Several times a day	3.91	1.34	11.41	0.01
Daily	3.30	1.13	9.63	0.03
Several times a week	2.76	0.93	8.18	0.07
Several times a month	2.94	0.96	9.06	0.06
**Unhealthy beverage consumption (ref: Less or never)**				
Several times a day	2.86	2.11	3.88	<0.001

Variables	OR	95% CI		P-value
		LCI	UCI	
Daily	2.02	1.52	2.68	<0.001
Several times a week	1.33	0.99	1.76	0.05
Several times a month	1.30	0.97	1.73	0.08
**Alcohol consumption**				
Yes	10.87	9.13	12.98	<0.001
**Physical activity (ref: Less)**				
Moderate	0.86	0.72	1.03	0.11
Vigorous	1.07	0.99	1.15	0.08

References

[1] World Health Organization, Ministry oh Health Republic of Indonesia. GYTS│GLOBAL YOUTH TOBACCO SURVEY FACT SHEET INDONESIA 2019. 2020.

[2] Beusenberg M, Orley J. A User's Guide to The Self Reporting Questionnaire (SRQ). 1994.

[3] Sheehan D, Janavs J, Baker R, Harnett-Sheehan K, Knapp E, Sheehan M, et al. M.I.N.I. Mini International Neuropsychiatric Interview English Version 5.0.0 DSM-IV. 2006.

[4] National Institute of Health Research and Development (NIHRD). Basic Health Research 2018 (Riskesdas 2018) National Report. Jakarta: 2019.

[5] Tjandrarini DH, Ashar H, Nari JP, Malakauseya MLV, Senewe FP, Musoddaq MA, et al. Disabilities and depression in young adolescents living in underdeveloped areas of Indonesia: Results from the 2018 Indonesia Basic Health Survey. BMJ Open 2025;15. https://doi.org/10.1136/bmjopen-2024-089737.

[6] World Health Organization. WHO steps surveillance manual: the WHO stepwise approach to chronic disease risk factor surveillance. WHO; 2005.

[7] Hartmann C, Siegrist M, Van Der Horst K. Snack frequency: Associations with healthy and unhealthy food choices. Public Health Nutrition 2013; 16:1487–96. https://doi.org/10.1017/S1368980012003771.

[8] World Health Organization. Global Physical Activity Questionnaire (GPAQ) Analysis Guide. Geneva: World Health Organization; 2021.
